# Editorial: Ultra-processed foods and human and planetary health

**DOI:** 10.3389/fnut.2023.1297262

**Published:** 2023-09-29

**Authors:** Gustavo Cediel, Raquel de Deus Mendonça, Adriana Lúcia Meireles, Maria Alvim Leite, Maria F. Gombi-Vaca, Fernanda Rauber

**Affiliations:** ^1^School of Nutrition and Dietetics, University of Antioquia, Medellín, Colombia; ^2^Department of Clinical and Social Nutrition, School of Nutrition, University of Ouro Preto, Ouro Preto, Brazil; ^3^Research and Teaching Group in Nutrition and Public Health, Graduate Program in Health and Nutrition, Federal University of Ouro Preto, Ouro Preto, Brazil; ^4^Department of Preventive Medicine, School of Medicine, University of São Paulo, São Paulo, Brazil; ^5^Center for Epidemiological Research in Nutrition and Health, University of São Paulo, São Paulo, Brazil; ^6^Rudd Center for Food Policy and Health, University of Connecticut, Hartford, CT, United States

**Keywords:** ultra-processed food, non-communicable diseases, sustainable, food environment, inequality, health

In 2010, the Nova food classification system introduced the definition of “ultra-processed” as a food category, and it has been adopted as a system to categorize foods to better understand the role of ultra-processing of foods on human and planetary health. Since then, Nova has been used in scientific studies and has been referenced in documents and recommendations released by national governments, international organizations, and civil society.

Ultra-processed foods (UPF) are defined as industrial formulations made by deconstructing whole foods into food-derived substances (e.g., fats, sugars, starch, protein isolate), altering them, and recombining them with additives, like colors, flavors, and emulsifiers into products (1). Typical examples of UPF are soft drinks, fast foods, chicken nuggets, instant soups, fruit drinks, and flavored yogurts. UPF are made and sold by transnational food corporations, and are convenient, affordable, and hyper-palatable. These foods displace fresh and minimally processed foods and freshly prepared meals and have occupied a significant portion of the diet in various populations (2).

This current Research Topic describes the impact of UPF on health outcomes and inequalities, and validates, even more, UPF as an indicator in diet-related studies.

Five articles focused on the effect of UPF specifically on health-related outcomes. Rudakoff et al. investigated the association between UPF consumption and adiposity in young Brazilian adults. This study found an association between UPF consumption and increases in body mass index (BMI), body fat percentage, fat mass index, android and gynoid fat, and decreases in lean mass percentage. Ashraf et al. evaluated the degree of food processing and its association with anthropometric measures among Canadian families with preschool-aged children, and found that consumption of UPF was positively associated with BMI and waist circumference in adults and children. Nilson et al. estimated cardiovascular disease premature deaths and incident cases, and the disability adjusted life-years attributable to UPF consumption among Brazilian adults, and found that ~22% of the premature deaths from cardiovascular disease and 33% of the total premature all cause deaths were attributable to UPF intake. Lopes et al. assessed the impact of food consumption, categorized by the degree of processing, on the serum fatty acid levels and lipid profiles of women with severe obesity. They observed an association between the consumption of processed and UPF and unfavorable lipid profiles and fatty acid levels among the participants. Finally, Coletro et al. described the association between co-occurrence of health risk behaviors (e.g., sedentarism, high frequency of UPF consumption, non-daily consumption of fruits and vegetables) and symptoms of anxiety and depression in adults. The study concluded that the combination of two and three health risk behaviors was associated with higher prevalence of the symptoms of anxiety or depression.

Two articles focused on consumption of UPF during pregnancy and in complementary feeding. Kelsey et al. described the association between UPF intake, diet quality, and dietary and inflammatory biomarkers among Norwegian women during mid-pregnancy. This study found that higher UPF intake was associated with reduced concentrations of nutrition biomarkers in mid-pregnancy. Moreira et al. conducted a randomized clinical trial to understand the association between different methods of food introduction (conventional technique/Parent-Led Weaning—PLW; Baby-Led Introduction to SolidS—BLISS); and mixed technique (both PLW and BLISS methods) and UPF consumption in early childhood. The study found that complementary feeding intervention focused on promoting infant autonomy (BLISS and mixed) was associated with reduction in the offer of UPF.

Two articles focused on the consumption of UPF and socioeconomic inequalities. Louzada et al. found that socioeconomic inequalities in food consumption decreased over a 10-year period in Brazil, but it may lead to the overall deterioration of the dietary quality for the more vulnerable populations. Crepaldi et al. examined the intersectionality of education, sex and race/skin color inequalities on consumption of unprocessed, minimally processed and UPF among Brazilians. The authors found that educational inequalities more strongly affected unprocessed/minimally processed food consumption than UPF. They also noted greater UPF inequalities among black/brown men and women than among white men.

Finally, there were two studies focused on validation of the Nova system included in this Research Topic. Huybrechts et al. used Nova classification to compare diets across the cultural and socio-economic diversity of European populations and validated it against biomarker measurements. Based on a large pan-European cohort, it demonstrated sociodemographic and geographical differences in the consumption of UPF. Furthermore, the results suggest that Nova classification can accurately capture UPF consumption, reflected by stronger correlations with food processing biomarkers (i.e., plasma elaidic acid, an unsaturated trans-fatty acid, and urinary 4-methylsyringol sulfate). Zancheta Ricardo et al. compared the frequency of UPF and their dietary share among the diet of Chilean preschoolers applying three distinct methods to identify UPF. The study found that searching for all possible markers of UPF in the list of ingredients increased the proportion of food products identified as UPF when compared to a classic method of food classification.

While this Research Topic did not yield published manuscripts specifically addressing the environmental impact of UPF, we strongly urge researchers to delve into this crucial aspect. UPF are typically associated with large-scale food production, which is often environmentally unsustainable. In the healthy and sustainability perspective, food extends beyond its mere nutritional components, encompassing a broader perspective that values health, supportiveness and sustainability. On one hand, this perspective promotes the adoption of dietary patterns based on natural foods, acquired through cooperative socio-environmental models that align with nature conservation and the unique culinary traditions of each region. On the other hand, it discourages dietary patterns associated with the corporate food industry, characterized by mass production of UPF. Such patterns have been associated with various forms of malnutrition and chronic diseases. Additionally, they are often entwined with marketing practices that tend to be socially unfair and environmentally unsustainable (3, 4). By centering our focus toward promoting more sustainable whole food options we will contribute to improve human health and to a more balanced relationship with our planet.

## Author contributions

GC: Writing—original draft. RM: Writing—review and editing. AM: Writing—review and editing. ML: Writing—review and editing. MG-V: Writing—review and editing. FR: Writing—review and editing.
